# Saint Louis Encephalitis Virus, Brazil

**DOI:** 10.3201/eid1301.060905

**Published:** 2007-01

**Authors:** Adriano Mondini, Izabela Lídia Soares Cardeal, Eduardo Lázaro, Silva H. Nunes, Cibele C. Moreira, Paula Rahal, Irineu L Maia, Célia Franco, Delzi V. N. Góngora, Fernando Góngora-Rubio, Eliana Márcia Sotello Cabrera, Luiz Tadeu Moraes Figueiredo, Flavio Guimarães da Fonseca, Roberta Vieira Moraes Bronzoni, Franscisco Chiaravalloti-Neto, Maurício Lacerda Nogueira

**Affiliations:** * Faculdade de Medicina de São José do Rio Preto, São José do Rio Preto, São Paulo, Brazil; † Secretaria Municipal de Saúde, São José do Rio Preto, São Paulo, Brazil; ‡ Universidade Estadual Paulista, São José do Rio Preto, São Paulo, Brazil; § Hospital de Base de São José do Rio Preto, São Paulo, Brazil; ¶ Universidade de São Paulo, Ribeirão Preto, São Paulo, Brazil; # Universidade Federal de Minas Gerais, Belo Horizonte, Minas Gerais, Brazil; 1 These authors contributed equally to this work.

**Keywords:** Saint Louis encephalitis virus, dengue, flavivirus, Brazil, letter

**To the Editor:** Saint Louis encephalitis virus (SLEV), a member of the *Flaviviridae* family, is widely dispersed in the Americas (*1**,**2*). In Brazil, SLEV was first isolated in the 1960s from a pool of mosquitoes at the Amazon Basin. Subsequently, the virus was repeatedly isolated from animals and arthropods in the Amazon region and São Paulo state (*3*). Nonetheless, isolation of SLEV from humans is rare; only 2 isolates from humans were described before 2005. Each isolate was from a patient who had jaundice and febrile illness without any neurologic symptoms (*1**,**3*). Recently in São Paulo, SLEV was isolated from a patient who had an incorrect diagnosis of dengue fever (*2**,**4*).

Despite the rare isolation of SLEV from humans, antibodies to this virus have been found in ≈5% of studied populations in the north and southeast regions of Brazil. However, because of antibody cross-reactivity among different flaviviruses and the fact that this population is vaccinated against yellow fever and exposed to dengue virus (DENV), such results should be interpreted carefully. Nevertheless, in these areas, SLEV may circulate and infect humans, although most infections are undiagnosed (*1**,**3**,**5*).

In contrast to previous instances in which the disease was detected in only 1 patient, we describe the first community outbreak of SLEV in Brazil. The outbreak was detected in São José do Rio Preto (population 400,000), in northwest São Paulo state. This outbreak was concurrent with a large outbreak of DENV serotype 3 (DENV-3), which occurred during the first half of 2006, with >15,000 possible cases reported to public health authorities. During this time, we were involved in an epidemiologic study to monitor the disease. We tested ≈250 samples for DENV, and 65% were positive. We tested for SLEV only those patients who were in our hospital or those who were referred to us for SLEV testing after an initial diagnosis of SLEV or DENV. The protocol approved by our ethical committee allowed us to test only samples from these patients (process no. 300/2004).

We used a multiplex nested reverse transcription–PCR (RT-PCR) assay to identify the most common flaviviruses in Brazil (DENV-1, DENV-2, DENV-3, yellow fever virus) as well as DENV-4, Ilheus virus, Iguape virus, Rocio virus, and SLEV. Of 54 samples (49 serum and 5 cerebrospinal fluid [CSF]) that were negative for DENV and yellow fever virus, SLEV RNA was detected in 6 (4 serum and 2 CSF) (*6*). RT-PCR results were negative for all other tested flaviviruses. Sequences of the amplified SLEV cDNAs from the 2 CSF samples were determined by using an ABI377 automated sequencer (Applied Biosystems, Foster City, CA, USA). The resulting sequences (GenBank accession nos. Q836336 and DQ836337) were identical and showed 96% homology to an Argentinean SLEV isolate (AY632544). All 6 SLEV-infected patients had an initial diagnosis of dengue fever or viral encephalitis; 3 had a diagnosis of viral meningoencephalitis, and the other 3 had signs of hemorrhagic disease (Table).

**Table:** Clinical data, 6 patients with Saint Louis encephalitis, Brazil, 2006*

Patient no. (age)	Sample tested by RT-PCR	Date of hospital admission	Initial diagnosis at admission	Signs, symptoms, selected laboratory results
1 (27 y)	Serum	Feb 25	Dengue fever	Clinical: fever, abdominal pain, diarrhea Serum: AST 58 IU/mL, ALT 69 IU/mL
2 (7 mo)	Serum	Mar 06	Dengue hemorrhagic fever, viral encephalitis	Clinical: fever, abdominal pain, melena, petechiae, positive tourniquet test Serum: platelets 311,000 /mm^3^, hematocrit 29% CSF: 13 cells/mm^3^, lymphocytes 86%, monocytes 14%
3 (37 y)	Serum	Apr 22	Dengue hemorrhagic fever	Clinical: fever, headache, chills, myalgia, maculopapular rash, positive tourniquet test Serum: hematocrit 43%, platelets 280,000/mm^3^ History: previous DENV infection (2002)
4 (34 y)	Serum	Apr 23	Dengue hemorrhagic fever	Clinical: fever, headache, chills, myalgia, maculopapular rash, positive tourniquet test Serum: platelets 141,000/mm^3^, hematocrit 38%, AST 81 IU/mL, ALT 56 IU/mL
5 (5 y)	CSF	Jun 05	Viral meningoencephalitis	Clinical: fever CSF: 286 cells/mm^3^, lymphocytes 60%, polymorphonuclear cells 37%, eosinophils 3%
6 (11 y)	CSF	Jun 07	Viral meningoencephalitis	Clinical: fever, facial palsy CSF: 12 cells/mm^3^, lymphocytes 100%

*RT-PCR, reverse transcription–PCR; AST, aspartate aminotransferase; ALT, alanine aminotransferase; CSF, cerebrospinal fluid; DENV, dengue virus.

Dengue is widely disseminated in Brazil and causes large outbreaks almost every year. The high prevalence of antibodies in the Brazilian population (*1*,*3*,*6*) suggests that SLEV infections are being misdiagnosed; its importance is underestimated. Brazil has no SLEV surveillance programs, and health professionals do not usually consider SLEV among their differential diagnoses. This SLEV outbreak was detected in a large urban center and was not specifically linked to patients who dwell in pockets of tropical forests, as previously reported (*1**–**4*).

This outbreak may represent the first time that hemorrhagic signs have been linked to SLEV infections. SLEV-associated hemorrhagic manifestations have not been reported in the literature. However, of our 6 SLEV-infected patients, 3 had hemorrhagic signs. Substantiating a causal link between SLEV infection and such clinical manifestations is difficult because DENV is endemic in the studied region (*7*). Possibly, SLEV-infected patients with hemorrhagic signs may have been previously infected by DENV. No reports have linked hemorrhagic manifestations to sequential DENV and SLEV infections; this possible link needs to be carefully evaluated.

In Argentina, SLEV has been isolated several times from animals (*8*). In some regions, SLEV seroprevalence in humans is ≈13% (*9*), but the number of documented human infections is small (*10*). These findings indicate either that SLEV is more prevalent than reported or that SLEV is reemerging. The Brazilian cases may parallel the situation in Argentina.

Our results clearly indicate an SLEV outbreak among this local population in Brazil. This outbreak differs from isolated infections previously described and indicates that this disease may be more prevalent in Brazil. In fact, the number of samples tested for SLEV during this DENV outbreak was relatively small. Had more samples been investigated, more cases of SLEV infection might have been found. A more comprehensive epidemiologic study is required to fully assess the magnitude of SLEV infection in Brazil.
